# Laparoscopic Repair of Colorectal Perforations Induced by Compressed Air Pressure: A Case Report

**DOI:** 10.7759/cureus.56007

**Published:** 2024-03-12

**Authors:** Ibrahim Elnogoomi, Hoorieh Qasemi, Mariam Aylan Alshamsi, Majid Alhammadi, Omar Elnogoomi

**Affiliations:** 1 General Surgery, Kuwait Hospital, Sharjah, ARE; 2 General Surgery, College of Medicine, University of Sharjah, Sharjah, ARE; 3 Surgical Oncology, National Cancer Institute, Cairo University, Cairo, EGY

**Keywords:** laparoscopy, colorectal perforation, compressed air, tension pneumoperitoneum, barotrauma, workplace injury

## Abstract

A compressed air nozzle has the potential to result in lethal injuries when handled inappropriately. Owing to the rarity of colorectal perforations due to barotrauma, no clear pathway to managing them has been established. We report an incident of a 33-year-old male patient who presented with tension pneumoperitoneum due to rectosigmoid perforations after being subjected to transanal compressed air insult. An emergency laparoscopic exploration with primary repair of the rectal perforation and Hartmann procedure were performed resulting in a smooth postoperative course. We hereby conclude that laparoscopy is a safe and effective approach associated with faster recovery and fewer adverse events.

## Introduction

The first ever documented case of colonic injury resulting from elevated air pressure can be traced back to 1904 by Dr. Stone, where the patient died. Later Andrews reported a second case of pneumatic rupture of the sigmoid colon, and Cotton reported a third case of pneumatic perforation in the ascending colon. These cases, occurring in 1911 and 1912, respectively, ended in recovery of the patients. Up until 2020, there has been a total of 53 accurately reported cases of rectal and colonic injuries secondary to the inappropriate use of industrial airs, in the English and Spanish literature, that were managed through laparotomy [1].

Colorectal perforations induced by compressed air pressure are rare but lethal because they can result in tension pneumoperitoneum. The substantial tension has the potential to cause fatal hemodynamic instability by compressing the inferior vena cava and splanchnic circulation, and respiratory compromise by elevating the diaphragm [2]. Pneumatic colorectal injuries caused by high-pressure compressed air can lead to perforations without the direct insertion of the air nozzle into the anus [3]. Such injuries, often resulting from accidental or purposeful use of compressed air in proximity to the perineum, highlight the need for awareness among workers regarding the potential for serious colorectal injuries [4]. The resting pressure of the anal sphincter ranges from 40 to 80 mm Hg and the pressure required to perforate the human gastrointestinal (GI) tract is around 213 mm Hg. Industrial air compressors are capable of delivering pressures that range from 7757 to 51,714 mm Hg, which are 36.4- to 242.7-fold greater than that required to perforate the GI tract [5,6]. In the context of surgical emergencies like colorectal perforations, the laparoscopic approach was less frequently used despite the lower complication rates and mortality associated with it compared to open surgery [7]. However, the safety and efficacy of laparoscopic interventions in the context of colonic perforation caused by compressed air have not been explored. In this report, we present a successful laparoscopic management of the bowel perforation caused by barotrauma from compressed air that, to our knowledge, has only been reported in the literature once [1].

## Case presentation

**Figure 1:**
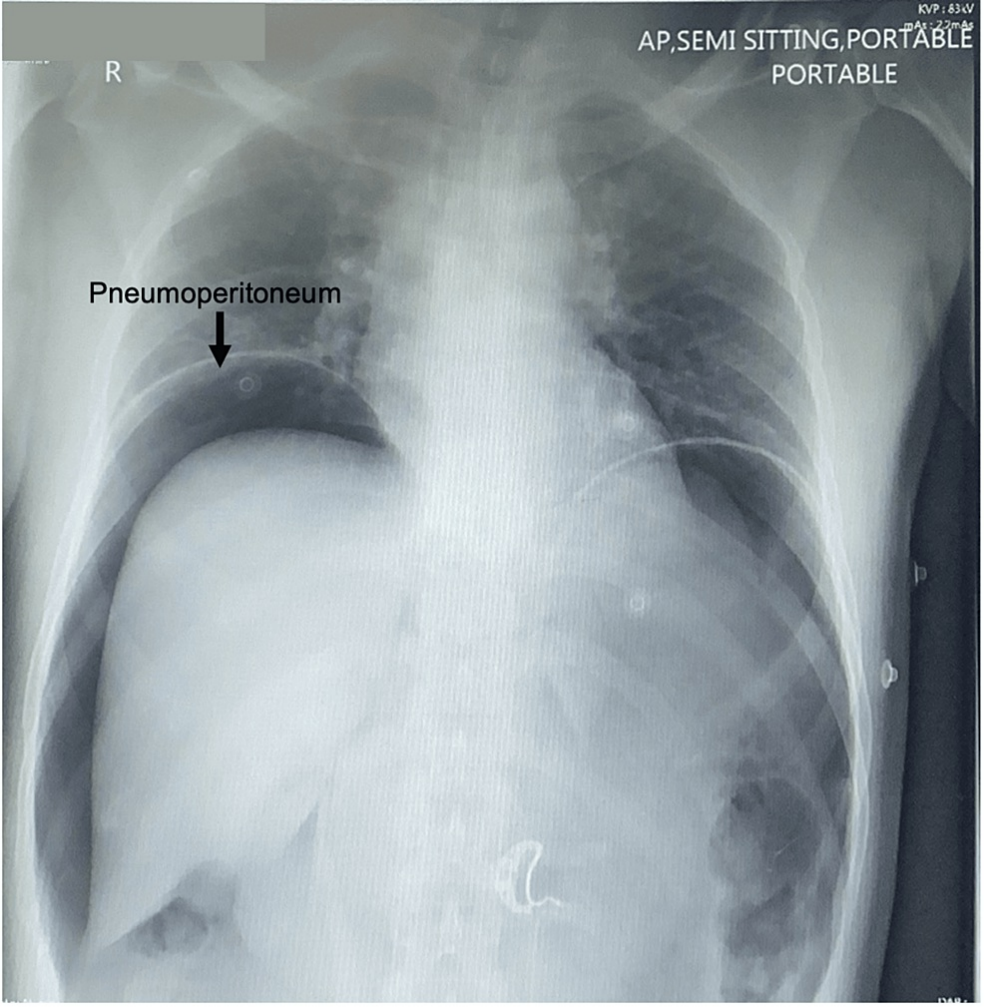
Semi-sitting portable chest X-ray showing bilateral marked pneumoperitoneum

**Figure 2:**
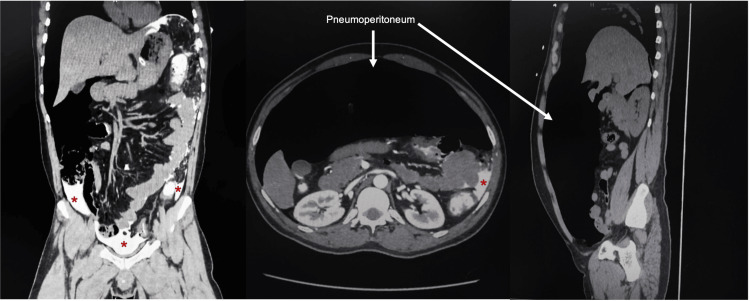
Abdomen CT with IV and rectal contrast Marked pneumoperitoneum in addition to rectal contrast extravasation at the rectosigmoid junction into the pelvis, paracolic gutter, and perisplenic region indicated by red asterisks.

**Figure 3:**
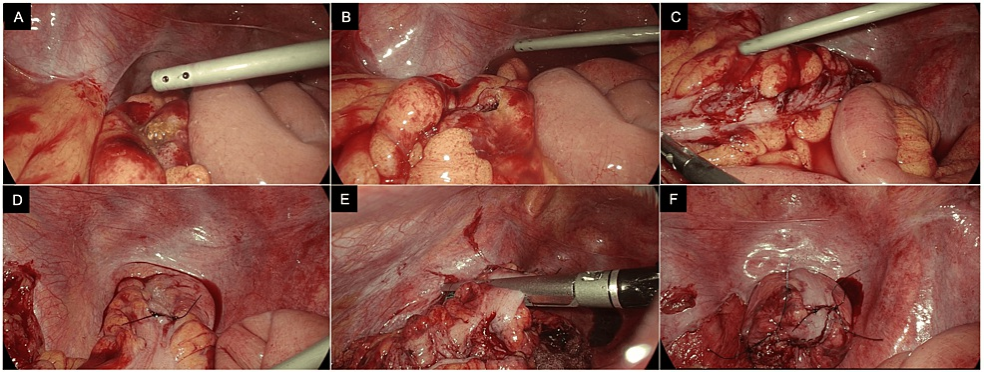
Intraoperative images (A) Sigmoid colon perforation with visible fecal matter. (B) Sigmoid colon perforation after irrigation. (C)Multiple serosal and seromuscular tears. (D) Distal most serosal tear after repair. (E) Laparoscopic stapler being used to resect non-viable segments. (F) Stitches were placed at the angles to support the staple line.

A 33-year-old Southeast Asian male was brought by the ambulance to the emergency department with an alleged history of being subjected to a compressed air insult at his workplace following which he developed severe abdominal pain and distension. As reported by the patient, he was washing a vehicle when his coworker jokingly placed the air compressor dust gun close to his anus over his clothes for less than a second. Immediately after that the patient fell on the floor, writhing in pain. At presentation, the patient was in apparent distress with a pulse rate of 61 beats/min, blood pressure of 145/105 mm Hg, respiratory rate of 24 breaths/minute, and an SpO_2_ of 96%. His abdomen was distended and rigid with diffuse tenderness and guarding. The external genitalia looked normal, and no perineal injuries or lesions were noted. The digital rectal examination was painful and revealed gross blood on the glove, but no defect or perforation was felt. Proctoscopy was not performed because of severe pain. A portable chest X-ray showed bilateral marked pneumoperitoneum (Figure 1). A CT scan of the abdomen with IV and rectal contrast confirmed the X-ray findings and showed evidence of rectal contrast extravasation at the rectosigmoid junction into the pelvis, paracolic gutter, and perisplenic region (Figure 2). The decision to attempt laparoscopic repair was taken. A 10-mm port was passed through the supraumbilical port site; the abdomen was deflated by the presumed camera port and then re-inflated up to 15 mm Hg by carbon dioxide. Three more ports were inserted: a 5-mm port in the left subcostal region, a 5-mm port in the right supraumbilical region, and a 10-mm port in the right infraumbilical region. On exploration, a 20-cm segment of the distal sigmoid and proximal rectum showed two perforations and multiple serosal and seromuscular tears. The pelvis, right and left paracolic gutters, and bilateral subphrenic spaces were soiled with fecal matter and blood (Figure 3). The distal most serosal tear was repaired, a segment 5 cm proximal to it measuring 15 cm consisting of the sigmoid colon and distal part of the descending colon was resected, and an end colostomy was done (Hartmann procedure) (Video 1). The rest of the colon and small intestine were examined laparoscopically, and no injuries were noted. He had a smooth postoperative course and was fit for discharge on the third postoperative day. The patient was offered follow-ups and Hartmann reversal, but he decided to continue treatment in his home country.

**Video 1:** Intraoperative video highlighting the defects seen on exploration and how they were managed

## Discussion

Barotrauma is an injury caused by a difference in the pressure between a gas inside, in contact with, or outside the body and the pressure of the surrounding gas or fluid. Damage results from overtension or sheer force from expansion of the gas within, or by pressure hydrostatically transmitted through, the tissues [8]. Most cases of colonic barotrauma reported in the literature describe iatrogenic insufflation during colonoscopy as the cause with rates ranging from 0.01% to 0.3% [9]. In the civilian setting, such injuries are reported among people using compressed-air-delivering tools as part of their daily jobs. Ignorance regarding the seriousness of the adverse events may lead to the misuse of these tools, recreationally, in the form of jokes or for sexual reasons, and result in potentially fatal outcomes [5,6,10,11]. There have been previously documented cases of colonic injuries even with the air nozzle held 15 cm away from the anus, over the clothes [10,11].

The colonic injuries associated with compressed air occur not only due to the enormous pressure delivered by these tools, but also due to the rate at which the pressure is delivered. A compressed air jet can deliver 141 L/min that is 96.5-folds greater than the maximum safe level of air flow used in colonoscopy [6]. At this rate, it takes less than one to two seconds to cause major damage in the human gastrointestinal tract. Injuries could range from “cat scratch tears” to full thickness perforations. The antimesenteric surface of the sigmoid colon is the most commonly injured site. The rectosigmoid junction is especially at risk since it has bilateral fixation by taenia coli that limits its mobility increasing its susceptibility to barotrauma [10,11]. This is consistent with the sites of injuries reported in our case.

One of the feared repercussions of colonic perforations is tension pneumoperitoneum, which is a rare phenomenon characterized by massive accumulation of air in the peritoneal cavity, which results in a sudden increase in intra-abdominal pressure [12]. This is considered a surgical emergency since it can lead to acute respiratory insufficiency and hemodynamic instability [11].

The open surgical approach was used in all the previously reported cases of colorectal perforation caused by compressed air. Concerns regarding the longer duration of laparoscopic surgery and risk of inadvertent injury to surrounding structures are the main factors that discourage the use of laparoscopic surgery in emergencies. More recently, it has been proven that the laparoscopic approach is safe and effective in the management of abdominal emergencies [13,14]. Certain emergencies lie in the indeterminate territory primarily due to the lack of evidence backing up the use of laparoscopy in such cases. Over the years, a growing body of evidence supporting the use of laparoscopy in surgical emergencies has emerged due to its safety, faster recovery times, and lower mortality rates associated [15]. Laparoscopic management in our case largely contributed to the smooth postoperative recovery and a short hospital stay. Such results would have been difficult to achieve if the patient underwent laparotomy.

Such incidents shed light on the importance of educating employees about the hazards associated with the tools they use in their workplace, in addition to raising awareness about the dangers of inappropriate use of these tools. Although incidents like the one we presented are rare, they could have devastating consequences.

## Conclusions

Colorectal injuries should be considered in individuals who have experienced perineal barotrauma. These patients face an elevated likelihood of developing tension pneumoperitoneum. Immediate surgical intervention with laparoscopy is considered safe for the management of such colorectal perforation incidents as demonstrated in the case presented here. Inappropriate handling of workplace tools subjects individuals to life-threatening injuries as seen in this case. Such workplace injuries highlight the need for raising awareness about the appropriate use of these tools and their potential hazards.
